# Hyperoside as a UV Photoprotective or Photostimulating Compound—Evaluation of the Effect of UV Radiation with Selected UV-Absorbing Organic Compounds on Skin Cells

**DOI:** 10.3390/ijms24129910

**Published:** 2023-06-08

**Authors:** Anna Moukova, Lukas Malina, Hana Kolarova, Robert Bajgar

**Affiliations:** 1 Department of Medical Biophysics, Faculty of Medicine and Dentistry, Palacký University Olomouc, Hnevotinska 3, 775 15 Olomouc, Czech Republic; anna.moukova01@upol.cz (A.M.); lukas.malina@upol.cz (L.M.); hana.kolarova@upol.cz (H.K.); 2 Institute of Molecular and Translational Medicine, Faculty of Medicine and Dentistry, Palacký University Olomouc, Hnevotinska 3, 775 15 Olomouc, Czech Republic

**Keywords:** phototoxicity, UV-absorbing compounds, astragalin, beta-carotene, 2,4-dihydroxybenzophenone, 2-hydroxy-4-methoxybenzophenone, hyperoside, 3-(4-methylbenzylidene)camphor, pachypodol, trans-urocanic acid

## Abstract

Ultraviolet (UV) radiation is a non-ionizing radiation, which has a cytotoxic potential, and it is therefore necessary to protect against it. Human skin is exposed to the longer-wavelength components of UV radiation (UVA and UVB) from the sun. In the present paper, we focused on the study of eight organic UV-absorbing compounds: astragalin, beta-carotene, 2,4-dihydroxybenzophenone, 2-hydroxy-4-methoxybenzophenone, hyperoside, 3-(4-methylbenzylidene)camphor, pachypodol, and trans-urocanic acid, as possible protectives of skin cells against UVA and UVB radiation. Their protective effects on skin cell viability, ROS production, mitochondrial membrane potential, liposomal permeability, and DNA integrity were investigated. Only some of the compounds studied, such as trans-urocanic acid and hyperoside, had a significant effect on the examined hallmarks of UV-induced cell damage. This was also confirmed by an atomic force microscopy study of morphological changes in HaCaT cells or a study conducted on a 3D skin model. In conclusion, hyperoside was found to be a very effective UV-protective compound, especially against UVA radiation. Commonly used sunscreen compounds such as 2,4-dihydroxybenzophenone, 2-hydroxy-4-methoxybenzophenone, and 3-(4-methylbenzylidene)camphor turned out to be only physical UV filters, and pachypodol with a relatively high absorption in the UVA region was shown to be more phototoxic than photoprotective.

## 1. Introduction

Humans are exposed to ultraviolet (UV) radiation on a daily basis as it is a part of the terrestrial radiation, i.e., the light emission from the Sun that reaches the Earth’s surface. UV radiation is essential for life, but exposure to UV radiation (once or repeatedly) leads to short-term or long-term effects. Short-term harms include erythema, skin pigmentation, immunological changes, or photosensitivity, while long-term harms include photodermatosis, skin ageing, or photocarcinogenesis.

Since the above-mentioned effects of UV radiation on the skin are not only non-cosmetic but can even be fatal, we tend to find a way to protect ourselves. Photoprotection consists of endogenous factors, avoiding direct sunlight, wearing appropriate clothes, and superficially or orally taken photoprotective compounds [1,2]. Photoprotective compounds are divided into two groups—primary photoprotective compounds are represented mainly by UV-filters, while secondary photoprotective compounds are biologically active molecules (actives) whose molecular mechanism lies in the prevention of biochemical and molecular consequences that occur in the skin after UV absorption [3].

Primary photoprotection occurs naturally in the skin (one of many endogenous photoprotective factors is trans-urocanic acid that absorbs UVB, and its photoisomerization to cis-urocanic acid contributes to photoprotection [4]) but can be enhanced using artificial primary photoprotection—sunscreens. These can be organic absorbers or inorganic blockers. Inorganic blockers reflect or scatter the UV radiation that reaches the skin while organic filters absorb short-wave, high-intensity UV radiation, excite to a higher energetic state, and then release the absorbed energy as heat. Inorganic filters are usually micropigments with 10–100 nm particles (such as ZnO or TiO_2_) [3]. Organic filters can absorb either UVA or UVB radiation. UVA absorbers are meradimate or benzophenones—oxybenzone (2-hydroxy-4-methoxybenzophenon), benzophenone 1 (2,4-dihydroxybenzophenone), or avobenzone. UVB radiation can be absorbed by 4-aminobenzoic acid derivatives, octyl salicylate, octocrylene, camphor derivatives such as enzacamene (3-(4-methylbenzylidene)camphor), or zinc acid esters [3,5,6].

As secondary photoprotective compounds antioxidants (vitamins, polyphenols), osmolytes or DNA repair enzymes are used [7]. The skin is equipped with a lot of antioxidants that are used to protect against oxidative stress involved in cellular respiration. These also act to quench reactive oxygen species (ROS) caused by UV radiation, but there are not enough of them to eliminate them completely [1]. However, the same antioxidants (such as lipophilic vitamin E or vitamin C derivatives) can be added to sunscreens to increase their effectiveness [3]. Other protective mechanisms of the skin could be studied and empowered (such as the ability of vitamin D to increase keratinocytes survival, increase DNA repair, and decrease cyclobutane pyrimidine dimers (CPD) production). Similar protective mechanisms have been developed in plants and some of the present compounds have a photoprotective effect also in the human skin—these can be polyphenols, monoterpenes, flavonoids, organosulfides, and indoles with their antimutagenic and anti-carcinogenetic effects [8].

Astragalin is a flavonoid that not only provides antioxidant protection but also regulates and modulates various molecular targets such as transcription factors, enzymes, kinases, cell adhesion proteins, apoptotic and anti-apoptotic proteins, and inflammatory cytokines [9]. Pachypodol and hyperoside are other natural flavonoids that support endogenous antioxidant mechanisms in the human body and thus also have the potential to act as photoprotective compounds [10,11]. Carotenoids (for example, beta-carotene) are plant pigments and effective antioxidants that neutralize singlet oxygen and peroxyradicals formed during photooxidative processes [12], but most of the studies have failed to convincingly demonstrate their photoprotective ability.

**Figure 1:**
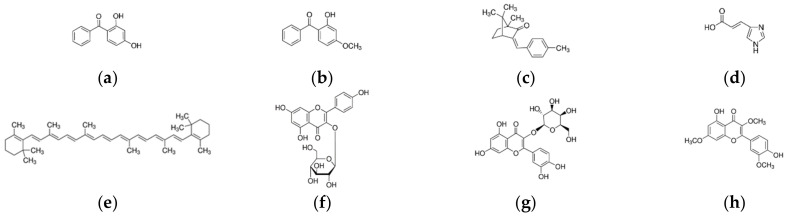
Chemical structure of the analyzed UV-absorbing compounds: (**a**) 2,4-dihydroxybenzophenone; (**b**) 2-hydroxy-4-methoxybenzophenone; (**c**) 3-(4-methylbenzylidene)camphor; (**d**) trans-urocanic acid; (**e**) beta-carotene; (**f**) astragalin; (**g**) hyperoside; (**h**) pachypodol.

The main goal of the presented work is to compare the protective potential of various groups of compounds against UVA and UVB radiation at the cellular level. In particular, our study evaluated the photoprotective effects of eight UV-absorbing compounds: astragalin, beta-carotene, 2,4-dihydroxybenzophenone, 2-hydroxy-4-methoxybenzophenone, hyperoside, 3-(4-methylbenzylidene)camphor, pachypodol, and trans-urocanic acid (Figure 1) on human keratinocytes HaCaT and epidermal model EpiDerm.

## 2. Results

### 2.1. Cytotoxicity and Phototoxicity of Selected UV-Absorbing Organic Compounds

**Figure 2:**
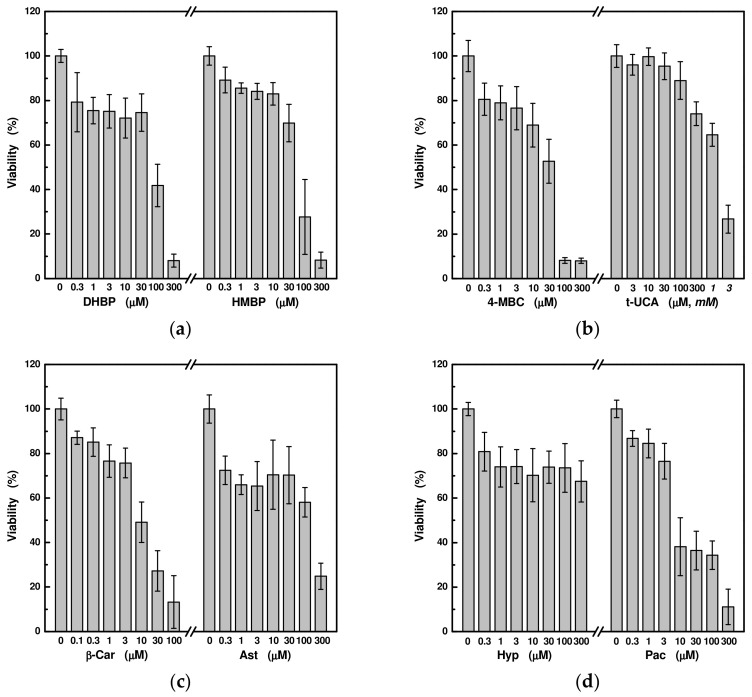
Cytotoxicity of selected UV-absorbing compounds: (**a**) 2,4-dihydroxybenzophenone (DHBP, IC50 = 85 μM) and 2-hydroxy-4-methoxybenzophenone (HMBP, IC50 = 56 μM); (**b**) 3-(4-methylbenzylidene)camphor (4-MBC, IC50 = 28 μM) and trans-urocanic acid (t-UCA, IC50 = 1.1 mM); (**c**) beta-carotene (β-Car, IC50 = 7.3 μM) and astragalin (Ast, IC50 = 78 μM); (**d**) hyperoside (Hyp, IC50 > 300 μM) and pachypodol (Pac, IC50 = 5.1 μM). Each value represents mean ± S.E. from 3 measurements.

Before evaluating the protection of eight UV-absorbing substances, we first determined the extent of their influence on cell proliferation in the HaCaT culture. Comparing the molar concentrations, pachypodol appeared to have the strongest negative effect on cell growth and division, causing more than a 50% reduction in cell viability even at 10 μM. On the contrary, trans-urocanic acid and hyperoside are the least toxic compounds, which even at 300 μM did not lead to the reduction below 60 % (Figure 2).

**Figure 3:**
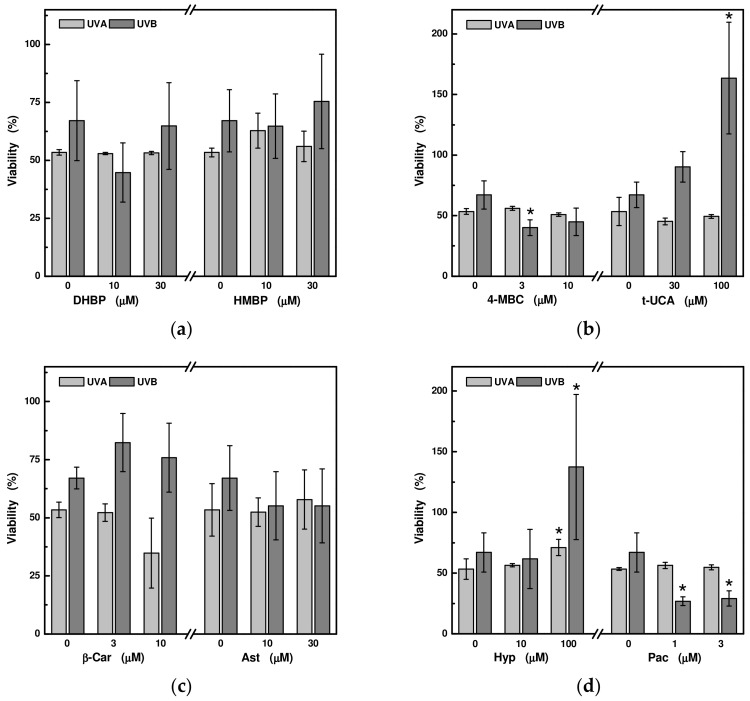
Relative changes in viability of HaCaT cells pre-treated with selected UV-absorbing compounds after exposure to UVA at a dose of 24 J/cm^2^ (i.e., at 6.8 mW/cm^2^ for 60 min) and UVB at a dose of 0.6 J/cm^2^ (i.e., at 3.5 mW/cm^2^ for 3 min): (**a**) 2,4-dihydroxybenzophenone (DHBP) and 2-hydroxy-4-methoxybenzophenone (HMBP); (**b**) 3-(4-methylbenzylidene)camphor (4-MBC) and trans-urocanic acid (t-UCA); (**c**) beta-carotene (β-Car) and astragalin (Ast); (**d**) hyperoside (Hyp) and pachypodol (Pac). The total yield of the MTT product by the control untreated cells was set as 100% viability. Each value represents mean ± S.E. from 3 measurements. * Significant difference compared to the sample without UV-absorbing compound (*p* < 0.05).

In the next study, HaCaT cells were incubated with two apparently non-toxic concentrations of these UV-absorbing compounds for 18 h and then their effect on the viability of HaCaT cells exposed to UVA (for 60 min at a dose of 24 J/cm^2^) and UVB radiation for 3 min (at a dose of 0.6 J/cm^2^) was monitored. We have previously reported that these irradiations themselves induce more than a 30% reduction in cell viability in this cell culture [13]. A statistically significant increase in cell viability was only observed in cells pre-treated with trans-urocanic acid and hyperoside at concentrations of 100 μM. While in the case of trans-urocanic acid, this increased proliferation was only linked with UVB radiation, in the case of hyperoside, this effect was also observed for UVA radiation, although to a lesser extent. For all other compounds, there was no significant change in cell viability except for pachypodol, where cell viability decreased significantly after UVB irradiation (Figure 3).

**Figure 4:**
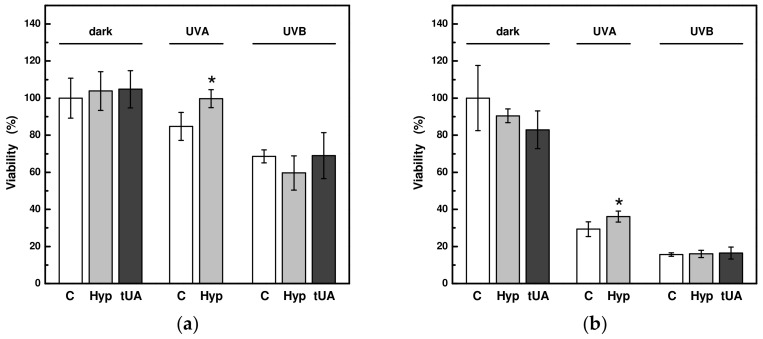
Relative changes in viability of epidermis model EpiDerm pre-treated with 30 μM hyperoside (Hyp) or 100 μM trans-urocanic acid (tUA) after exposure to UVA (at 6.8 mW/cm^2^) and UVB (at 3.5 mW/cm^2^) radiation: (**a**) at the 18 h pre-incubation with the UV-absorbing compounds and following irradiation with UVA at a dose of 24 J/cm^2^ or UVB at a dose of 3.1 J/cm^2^ and evaluation of the viability after 18 h from irradiation; (**b**) at the 42-h pre-incubation with the UV-absorbing compounds and following irradiation with UVA at a dose of 36 J/cm^2^ or UVB at a dose of 4.2 J/cm^2^ and evaluation of the viability after 4 days from irradiation. Control samples without the presence of the compounds are marked as C. Each value represents mean ± S.E. from 3 measurements. * Significant difference compared to the sample without UV-absorbing compound (*p* < 0.05).

This increased effect of trans-urocanic acid and hyperoside in combination with UV radiation on cell culture viability was verified on an epidermal skin model during 18 h or 42 h of incubation with these compounds. The results of these measurements confirmed that pre-treatment of the 3D skin model EpiDerm with hyperoside and subsequent UVA irradiation significantly increased its viability. However, no similar effect was observed for trans-urocanic acid (Figure 4).

### 2.2. ROS Production

**Figure 5:**
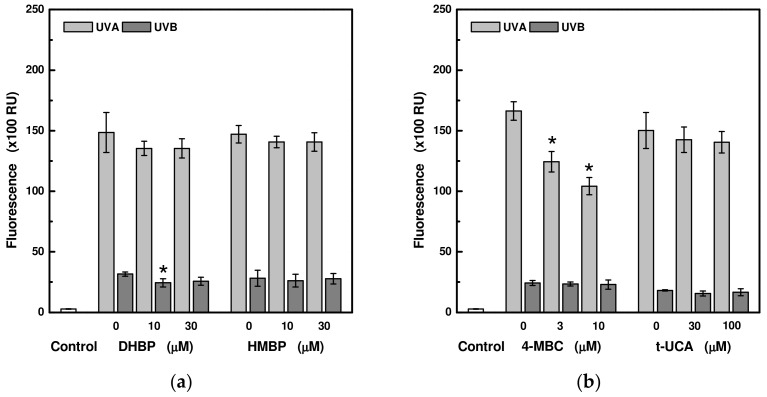

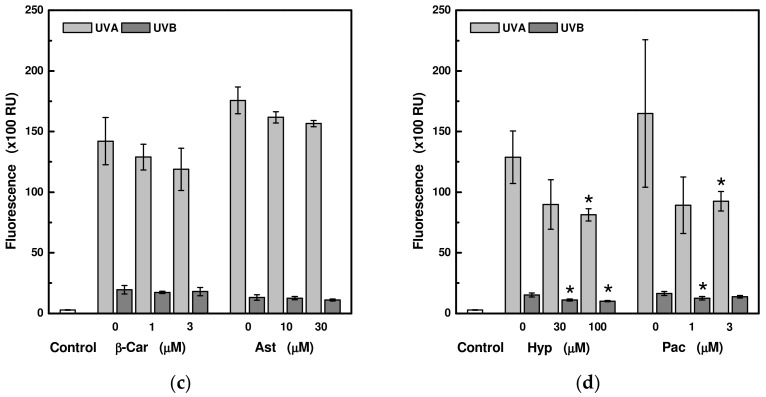
ROS production in HaCaT cells pre-treated with selected UV-absorbing compounds after exposure to UVA (at 24 J/cm^2^) and UVB (at 0.6 J/cm^2^) radiation: (**a**) 2,4-dihydroxybenzophenone (DHBP) and 2-hydroxy-4-methoxybenzophenone (HMBP); (**b**) 3-(4-methylbenzylidene)camphor (4-MBC) and trans-urocanic acid (t-UCA); (**c**) beta-carotene (β-Car) and astragalin (Ast); (**d**) hyperoside (Hyp) and pachypodol (Pac). Each value represents mean ± S.E. from 3 measurements. Control sample is a sample in the absence of the UV-absorbing compound and without irradiation. * Significant difference compared to the irradiated sample without UV-absorbing compound (*p* < 0.05).

The results of measuring ROS production in HaCaT cells pre-treated with UV-absorbing compounds and exposed to UVA or UVB radiation are shown in Figure 5. Pre-treatment of the cells with the investigated compounds at non-toxic concentrations did not lead to changes in the ROS production in the cells after irradiation with a UVA or UVB source for most of the studied compounds. An exception was the incubation of cells with hyperoside and 3-(4-methylbenzylidene)camphor. The treatment of cells with 100 μM hyperoside or treatment of cells with 3 and 10 μM 3-(4-methylbenzylidene)camphor led to a statistically significant decrease in ROS production during 60 min (24 J/cm^2^) of exposure to UVA radiation. Moreover, hyperoside at concentrations of 30 and 100 μM was also effective in reducing ROS induced by 3 min (0.6 J/cm^2^) of UVB irradiation (Figure 5d).

### 2.3. Changes in Mitochondrial Membrane Potential

**Figure 6:**
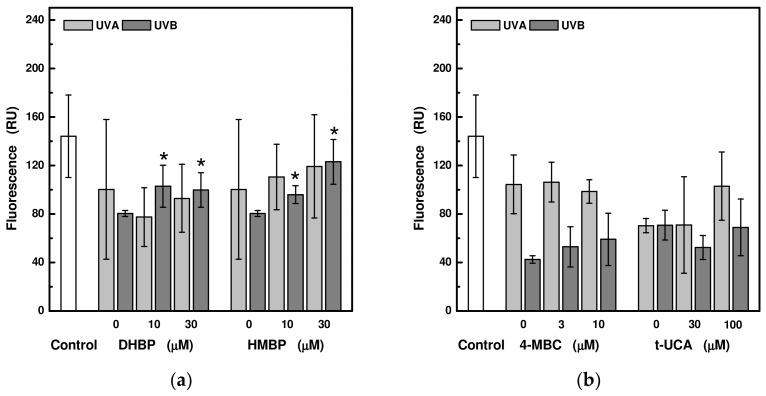

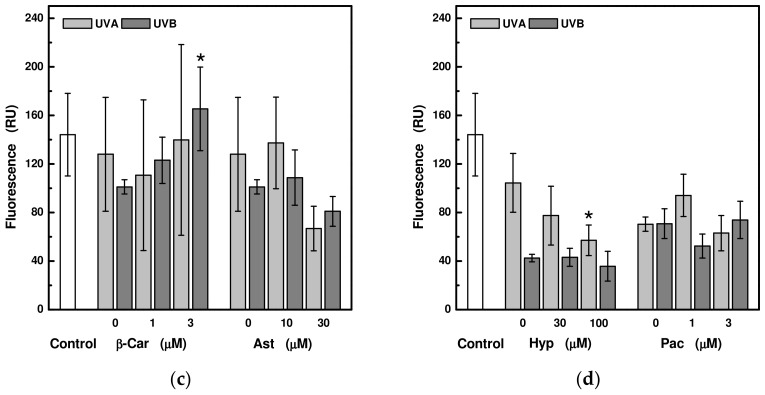
Relative changes in mitochondrial membrane potential in HaCaT cells pre-treated with selected UV-absorbing compounds after exposure to UVA (at 24 J/cm^2^) and UVB (at 1.2 J/cm^2^) radiation: (**a**) 2,4-dihydroxybenzophenone (DHBP) and 2-hydroxy-4-methoxybenzophenone (HMBP); (**b**) 3-(4-methylbenzylidene)camphor (4-MBC) and trans-urocanic acid (t-UCA); (**c**) beta-carotene (β-Car) and astragalin (Ast); (**d**) hyperoside (Hyp) and pachypodol (Pac). Each value represents mean ± S.E. from 3 measurements. Control sample is a sample in the absence of the UV-absorbing compound and without irradiation. * Significant difference compared to the irradiated sample without UV-absorbing compound (*p* < 0.05).

In order to find out whether the investigated UV-absorbing compounds prevent damage to the mitochondria of HaCaT cells as a result of the action of UV radiation, which is associated with a change in the mitochondrial membrane potential, the fluorescent probe JC-1 was used. Although the measurements are burdened by high deviations in the measured values, it turns out that the effect of 2,4-dihydroxybenzophenone, 2-hydroxy-4-methoxybenzophenone, and beta-carotene in a concentration-dependent manner leads to higher values of the mitochondrial membrane potential, whereas the effect of hyperoside, on the other hand, has the opposite effect (Figure 6).

### 2.4. Analysis of Lysosomal Membrane Integrity

**Figure 7:**
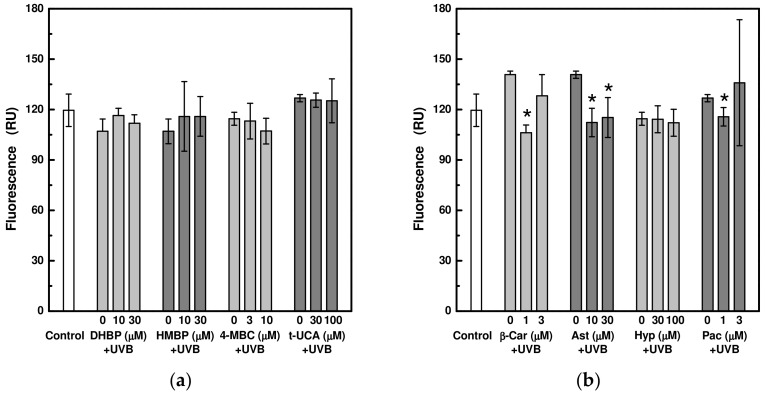
Determination of lysosomal membrane integrity in HaCaT cells pre-treated with selected UV-absorbing compounds after exposure to UVB (at 1.2 J/cm2) radiation: **(a)** 2,4-dihydroxybenzophenone (DHBP), 2-hydroxy-4-methoxybenzophenone (HMBP), 3-(4-methylbenzylidene)camphor (4-MBC), and trans-urocanic acid (t-UCA); **(b)** beta-carotene (β-Car), astragalin (Ast), hyperoside (Hyp), and pachypodol (Pac). Each value represents mean ± S.E. from 3 measurements. Control sample is a sample in the absence of the UV-absorbing compound and without irradiation. * Significant difference compared to the irradiated sample without UV-absorbing compound (*p* < 0.05).

Due to the strong UVA-induced photobleaching of the LysoTracker Blue DND-22 probe used (Figure S1), the effect of selected UV-absorbing compounds on the lysosomal membrane integrity of HaCaT cells exposed only to UVB radiation was studied. In most cases, the UV protective compounds used at non-toxic concentrations had no effect on the integrity of the lysosomes as a result of exposure to UVB radiation at an exposure dose of 1.2 J/cm^2^ (Figure 7).

### 2.5. DNA Damage

**Figure 8:**
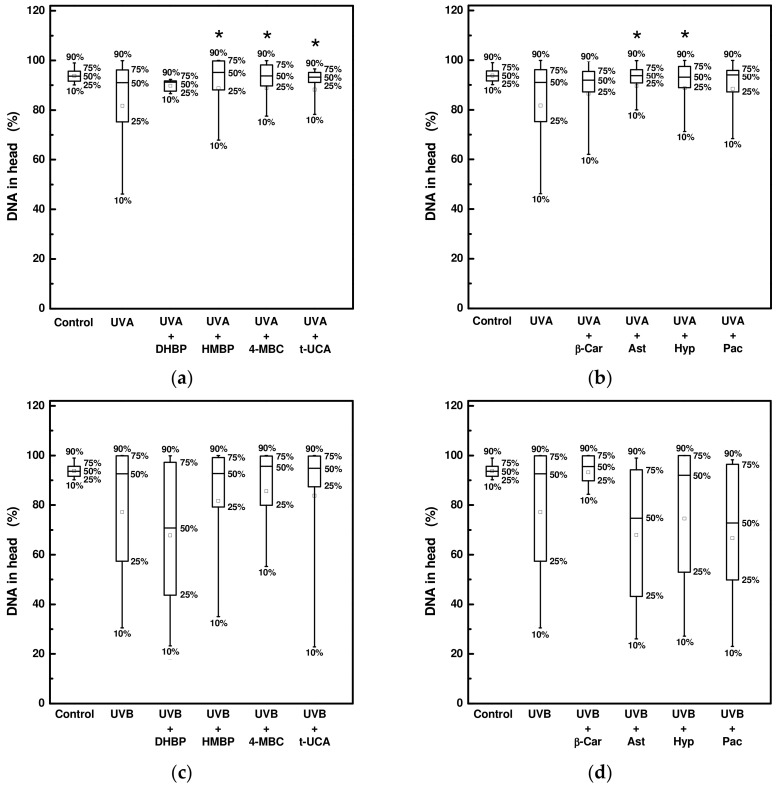
DNA fragmentation in HaCaT cells pre-treated with selected UV-absorbing compounds after exposure to: (**a**,**b**) UVA radiation (at 36 J/cm^2^); (**c**,**d**) UVB radiation (at 1.7 J/cm^2^). DHBP is 2,4-dihydroxybenzophenone at 30 μM, HMBP is 2-hydroxy-4-methoxybenzophenone at 30 μM, 4-MBC is 3-(4-methylbenzylidene)camphor at 10 μM, t-UCA is trans-urocanic acid at 300 μM, β-Car is beta-carotene at 3 μM, Ast is astragalin at 100 μM, Hyp is hyperoside at 300 μM, and Pac is pachypodol at 3 μM. Each box plot represents the 10th-, 25th-, 50th-, 75th-, and 90th-percentile determined from approximately 100 cells in an average. Control sample is a sample in the absence of the UV-absorbing compound and without irradiation. * Significant difference compared to the irradiated sample without UV-absorbing compound (*p* < 0.05).

The extent of DNA damage in HaCaT cells was determined using the Comet assay as previously described by Manisova and her co-workers [14]. The effect of eight UV-absorbing compounds at one of the higher non-toxic concentrations with UVA and UVB radiation was studied. When cells were irradiated with UVA radiation for 90 min (36 J/cm^2^), a reduction in DNA fragmentation, i.e., an increased percentage of DNA localized in comet heads, was recorded for cells pre-treated with 2-hydroxy-4-methoxybenzophenone, 3-(4-methylbenzylidene)camphor, trans-urocanic acid, astragalin, and hyperoside. In the case of UVB radiation for 8 min (1.7 J/cm^2^), none of the applied UV-absorbing compounds led to a statistically significant decrease in DNA fragmentation (Figure 8).

### 2.6. Changes in Cell Morphology

**Figure 9:**
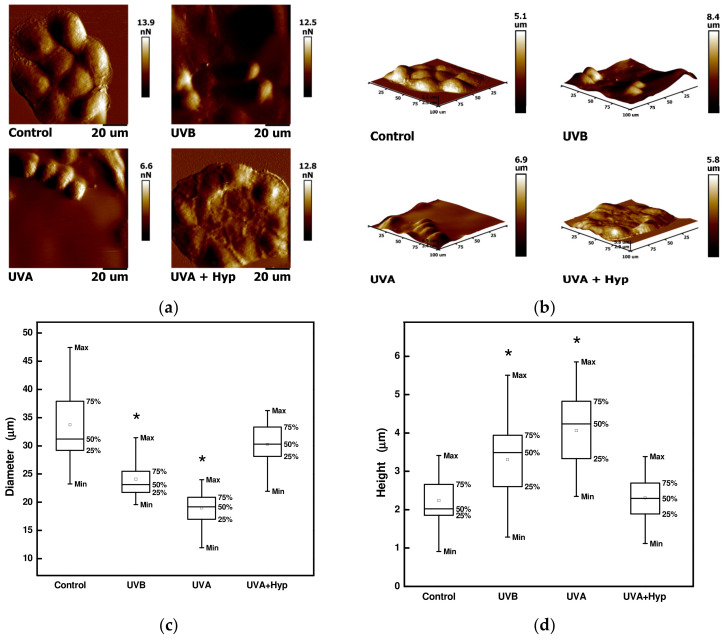
Atomic force microscopy of HaCaT cells pre-treated with 50 μM hyperoside (Hyp) for 4 h after exposure to UVA (at 36 J/cm^2^) and UVB (at 1.7 J/cm^2^) radiation: (**a**) peak force error images; (**b**) height images; (**c**) statistical analysis of the diameter of the cells; (**d**) statistical analysis of the height of the cells. Each box plot represents the minimum, first quartile, median, third quartile, and maximum determined from 17–21 cells. * Significant difference compared to the sample without pre-treatment with hyperoside and exposure to UV radiation (*p* < 0.05). Scans were acquired in a Peak Force tapping mode, at scan rate of 0.2–0.3 Hz, and image resolution of 256 × 256 pixels.

The size of HaCaT cells acquired by an atomic force microscope was calculated from 20 cells in an average using the NanoScope Analysis software. Cell diameter was measured along the longest part of the cell. Cells were first incubated with 50 µM hyperoside for 4 h and then irradiated with UVA radiation. The control cell scans (cells without any treatment) showed an irregular elongated shape with a median of the height of 2.02 μm and a median of the diameter of 31.21 μm. Irradiating the cells by UV led to cluster disruption and changes in shape. It caused cell swelling, resulting in a decrease in the diameter (19.16 μm for UVA; 23.13 μm for UVB), an increase in the height (4.24 μm for UVA; 3.44 μm for UVB), and an overall elliptic shape compared to control cells (Figure 9). However, the pre-treatment of the cells with hyperoside prevented cells from those UVA-induced morphological changes. After the irradiation, the cells still formed clusters and there were no significant changes in the median of the diameter (29.87 μm) and median of the height (2.24 μm).

## 3. Discussion

The presented paper studies the protective effect of eight UV-absorbing compounds on skin cells exposed to UVA and UVB radiation. The UVA component of the solar radiation falling on the Earth’s surface is significantly predominant (10 times or more than UVB [15]) and reaches irradiance values of about 3–6 mW/cm^2^ [16,17] during a sunny, summer day at the high solar zenith. In addition, the UVB component is particularly significant at midday between 10 am and 2 pm. For our measurements, we used UVA radiation with an irradiance value of 6.8 mW/cm^2^. In the case of UVB radiation, the irradiance value was higher than would correspond to solar radiation, but shorter exposure times were applied, in units of minutes. 2,4-Dihydroxybenzophenone, 2-hydroxy-4-methoxybenzophenone, 3-(4-methylbenzylidene)camphor, and trans-urocanic acid were selected as UV-absorbing compounds that show significant absorption in the UVB region, while beta-carotene, astragalin, hyperoside, and pachypodol also show substantial absorption in the UVA region (Figure S2). 2,4-Dihydroxybenzophenone, 2-hydroxy-4-methoxybenzophenone, and 3-(4-methylbenzylidene)camphor are common ingredients that are used in sunscreens as UV-filters. Trans-urocanic acid is a UVB chromophore normally found in human skin. Its exposure to UVB leads to photoisomerization, from the trans form to the cis form, thereby contributing to photoprotection [4]. Beta-carotene is a red-orange pigment found in colorful plants, fruits, and vegetables, especially in carrots. It is converted to vitamin A in animals and it is also known for its strong antioxidant properties. Astragalin is a natural flavonoid found in several medicinal plants such as *Cuscuta chinensis* [18]. It has already been studied for its anti-inflammatory, antioxidant, neuroprotective, cardioprotective, anti-obesity, anti-osteoporotic, anticancer, antiulcer, and antidiabetic properties [9]. Hyperoside is a flavonol glycoside present in plants, fruits, and vegetables. It also has some significant pharmacological actions, including anti-inflammatory, antithrombotic, antidiabetic, hepatoprotective, and antioxidant effects [19]. Pachypodol is a plant flavonol with antibacterial and antifungal activities [20].

In our previous work, we found that UVA or UVB radiation with the above-mentioned and applied irradiances caused a 30% decrease in the viability of HaCaT cells after 60 min of exposure to UVA or 3 min of exposure to UVB radiation, which was also associated with an increase in ROS production, and a decrease in mitochondrial membrane potential and DNA damage in the sense of its increased fragmentation [13]. Now, our data showed that an increase in viability only occurred in cells pre-treated with trans-urocanic acid and hyperoside. While in the case of trans-urocanic acid, this increased viability was only linked with UVB radiation, in the case of hyperoside, this effect was also observed for UVA radiation. This may be related to the fact that the acid does not show significant absorption in the UVA region, and therefore its potential of protection against UVA radiation may be negligible. In addition, it has been shown that a higher concentration of these compounds (100 μM), especially in combination with UVB radiation, increases the viability or even the proliferation of these cells, since there was a relative increase in the activity of the oxidoreductases involved in the MTT viability test, above the above-mentioned 30 %, which approximately corresponds to the extent of the harmful effect of the UV radiation components themselves. In the case of trans-urocanic acid, it is an interesting result, since it has already been published that the cis-form (which is formed during UVB irradiation) leads to the up-regulation of 16 genes in primary human keratinocytes, which are associated with apoptosis, cell growth arrest, and cytokine production [21]. However, it should be mentioned that this finding was associated with high concentrations of the cis form (72 μM and above), and no significant changes in gene expression occurred when keratinocytes were incubated with the trans-urocanic acid at the same concentrations. A significant increase in the viability of skin cells caused by hyperoside in combination with UVA radiation was also confirmed in the 3D epidermal model. A similar positive effect of hyperoside in a dose-dependent manner (5–100 μM) on cell viability has been reported for primary melanocytes subjected to oxidative stress by H_2_O_2_ [22]. On the other hand, it has been shown that hyperoside alone significantly inhibits the proliferation of malignant tumor cells [23,24,25]. The presence of hyperoside for 24 h halved the viability of cells A549 (lung adenocarcinoma), H1975 (non-small cell lung cancer), and PANC-1 (pancreatic cancer) at concentrations of around 400 μM [23], 100 μM [24], and 550 μM [25], respectively.

UV radiation generates ROS, including superoxide anions, singlet oxygen, hydroxyl radicals, and hydrogen peroxide, through various mechanisms, e.g., by affecting the enzyme catalase, up-regulating nitric oxide synthase synthesis, or via endogenous chromophores that can be damaged or act as photosensitizers, both leading to the ROS production [26]. Our measurements showed that only the presence of 3-(4-methylbenzylidene)camphor or hyperoside can reduce the ROS level in irradiated HaCaT cells. While hyperoside was effective for both UVA and UVB radiation, camphor significantly reduced the amount of ROS only in cells exposed to UVA radiation. Although the use of UV-filters in sunscreens is considered safe, there are several reports showing that some UV-filters penetrate through the stratum corneum and can enhance the ROS generation under UV radiation [27,28,29]. Nevertheless, our measurements regarding both studied benzophenones under the set conditions (at the concentrations of 10 and 30 μM, and radiation dose of 24.5 J/cm^2^ for UVA and 0.6 J/cm^2^ for UVB) did not show any differences in the production of ROS in HaCaT cells. In the case of beta-carotene, Lohan and her coworkers have already shown that, in the same cells exposed to higher doses of visible and infrared radiation inducing and forming ROS, beta-carotene at a concentration of 0.2 μg/mL also did not lead to a reduction in ROS [30]. In contrast, the antioxidant effect of hyperoside is already known. Hyperoside scavenged the intracellular ROS in hepatic stellate LX-2 cells [31], lung fibroblast V79-4 cells [32], and pheochromocytoma PC12 cells [33].

UV radiation is associated with a decrease in the mitochondrial membrane potential of HaCaT cells [13,34]. However, measurements with cells pre-treated with 2,4-dihydroxybenzophenone, 2-hydroxy-4-methoxybenzophenone, and beta-carotene showed higher values of the mitochondrial membrane potential after UVA or UVB irradiation. Of the benzophenones, 2-hydroxy-4-methoxybenzophenone (also known as benzophenone 3) is especially suspected to be an endocrine disruptor [35]. It has been demonstrated that it activates the apoptosis in mouse neuronal cells via an intrinsic pathway involving the loss of mitochondrial membrane potential [36]. Beta-carotene is considered as a nutrient with an antioxidant activity that is able to quench singlet oxygen in skin, and thus protect it against harmful UV radiation. However, there are some studies conducted on skin cells that revealed that beta-carotene not only acts as an antioxidant but also has prooxidant potential, especially if its breakdown products are taken into account [37,38,39]. While beta-carotene pre-treatment alters the effects of H_2_O_2_-induced damage in erythro-myeloblastoid leukemia K562 cells [40], its metabolites such as retinal or retinoic acid causes oxidative stress with a decrease in mitochondrial membrane potential [38,39]. In contrast to other studied UV-absorbing compounds, hyperoside, which increases the viability of skin cells exposed to UV radiation and reduces ROS production, led to an even significantly greater depolarization of the mitochondrial membrane potential than that induced by UV radiation alone. Hyperoside at higher concentrations possess antitumor activity [23,24,25] that can be triggered via ROS/NF-κB, ROS/p38 MAPK, and Ca^2+^/mitochondrion apoptotic signaling pathways with an alteration in mitochondrial membrane potential [25,41,42,43].

Photons of UVA radiation have lower energy but have the ability to penetrate deeper into the skin. In contrast to UVB, UVA is poorly absorbed by DNA but excites numerous endogenous chromophores, generating ROS through which DNA is damaged [26,44]. Our results showed that none of the studied compounds at non-cytotoxic concentrations significantly reduced the DNA fragmentation in cells exposed to UVB radiation. On the other hand, 2-hydroxy-4-methoxybenzophenone, 3-(4-methylbenzylidene)camphor, trans-urocanic acid, astragalin, and hyperoside appears to be the compounds that protect DNA during UVA radiation. Hyperoside was also included in a microscopic study aimed at the morphological changes of HaCaT cells exposed to the UVA and UVB radiation. Additionally, here, the beneficial effect of this compound was demonstrated. It contributed to the preservation of cell cohesion and the normal shape of cells.

## 4. Materials and Methods

### 4.1. Cell Culture, Epidermal Model, UV Irradiation, and Chemicals

The immortalized human keratinocytes HaCaT and reconstituted three-dimensional human epidermis model (EpiDerm, MatTek Europe, SVK) were grown in high-glucose DMEM (Sigma-Aldrich, Burlington, MA, USA) supplemented with sodium bicarbonate (3.7 g/L, Sigma-Aldrich, Burlington, MA, USA), fetal bovine serum (10% *v*/*v*, Biowest, France), and penicillin and streptomycin (100 U/mL and 100 μg/mL, respectively, Sigma-Aldrich, Burlington, MA, USA) in darkness under a humidified atmosphere with 5% CO_2_ at 37 °C. For all experiments, HaCaT was dissociated using TrypLE Express (Gibco by Thermo Fisher Scientific, Waltham, MA, USA) and transferred into 12-well plates at a total number of 1 × 10^5^ cells and cultivated for 24 h. The UV sources with relatively homogeneous irradiance were made from commercially available phototherapeutic UV tubes [13]. The UVA source had a peak maximum at 370 nm and FWHM of 25 nm, while the UVB source had a peak maximum at 310 nm and FWHM of 6 nm (Figure S2). The irradiance values were 6.8 ± 0.4 mW/cm^2^ for the UVA source and 3.5 ± 0.4 mW/cm^2^ for the UVB source. All UV-absorbing compounds tested (astragalin CAS No. 480-10-4, beta-carotene CAS No. 7235-40-7, 2,4-dihydroxybenzophenone CAS No. 131-56-6, 2-hydroxy-4-methoxybenzophenone CAS No. 131-57-7, hyperoside CAS No. 482-36-0, 3-(4-methylbenzylidene)camphor CAS No. 36861-47-9, pachypodol CAS No. 33708-72-4, and trans-urocanic acid CAS No. 104-98-3) were purchased from Sigma-Aldrich (Burlington, MA, USA) (Figure 1 and Figure S3).

### 4.2. Cytotoxicity and Phototoxicity Test

HaCaT cells and Epiderm were incubated for 18 h in DMEM supplemented with UV-absorbing compounds. Irradiation of the culture cells was performed in 0.6 mL/well phosphate-buffered saline (PBS) supplemented with 5 mM glucose (PBS-G) in 12-well plates. In the case of the 3D epidermal models, for irradiation, 3 inserts were always placed in one well of a 6-well plate filled with 0.9 mL of PBS-G; otherwise, they were kept separately in a 24-well plate with 0.3 mL of DMEM per well. After irradiation, PBS was replaced with fresh DMEM and the plates with cells were placed in a CO_2_ incubator for 18 h. The MTT assay was initiated by adding 5 mg/mL of methylthiazol tetrazolium bromide (MTT, Sigma-Aldrich) to DMEM, resulting in a 10-fold lower concentration of MTT in each well. After 3 h, the medium was completely removed and the formazan crystals produced by the cells were dissolved in 0.6 mL of DMSO for cell cultures or in 2 mL of isopropanol for epidermal models. The absorbance of these solutions was spectroscopically measured at 570 nm. The relative number of viable (oxidoreductase active) cells in the treated and untreated samples (controls) was expressed as a percentage.

### 4.3. ROS Measurement

The production of the intracellular ROS induced by UVA or UVB after 18 h of incubation with UV-absorbing compounds was analyzed using the fluorescence probe 5-(and-6)-chloromethyl-2′,7′-dichlorodihydrofluorescein diacetate (CM-H_2_DCFDA, Invitrogen by Thermo Fisher Scientific, Waltham, MA, USA). The HaCaT cells were incubated in PBS-G buffer supplemented with 10 μM CM-H_2_DCFDA for 20 min and then, after washing twice in PBS-G, exposed to UVA or UVB radiation for 60 or 3 min. After irradiation, the fluorescence of CM-DCF was recorded using a Tecan Infinite 200 pro fluorescence reader (Tecan, Männedorf, Switzerland) at excitation and emission wavelengths 490 nm and 525 nm, respectively.

### 4.4. Mitochondrial Membrane Potential Measurement

The mitochondrial membrane potential in HaCaT cells was estimated using the fluorescent probe 5,5′,6,6′-tetrachloro-1,1′,3,3′ tetraethylbenzimidazolylcarbocyanine chloride (JC-1, Biotium, Fremont, CA, USA). After UV irradiation in PBS-G, JC-1 was added to the cells at the final assay concentration of 2 μg/mL and the cells were allowed to incubate for 20 min. Then, the fluorescence emission intensity was measured using a Tecan Infinite 200pro fluorescence reader at excitation and emission wavelengths of 490 nm and 590 nm, respectively.

### 4.5. Lysosomal Membrane Integrity Measurement

Lysosomal membrane damage in HaCaT cells was determined using the acidotropic probe LysoTracker Blue DND-22 (Invitrogen by Thermo Fisher Scientific, Waltham, MA, USA) whose fluorescence depends on pH. HaCaT cells were incubated with 400 nM LysoTracker Blue DND-22 in PBS-G, then washed twice with PBS-G, and subsequently irradiated with UVB for 6 min. Due to the strong photobleaching effect of this probe caused by UVA radiation, the cells were not exposed to UVA radiation (Figure S1). The fluorescence intensity of LysoTracker Blue DND-22 with an excitation wavelength of 380 nm and an emission wavelength of 422 nm was measured using the Tecan Infinite 200pro fluorescence reader.

### 4.6. Comet Assay

DNA damage caused by UVA or UVB radiation to the HaCaT cells pre-treated with UV-absorbing compounds was investigated by a comet assay. The growth medium was replaced by PBS-G and the cells were irradiated by means of the UVA or UVB sources for 90 or 8 min, respectively. After irradiation, PBS-G medium was replaced with DMEM and the cells were allowed to culture for 24 h. The Comet assay was performed as previously described by Manisova and her co-workers [14]. The samples were finally stained by SYBR Green (Invitrogen, Thermo Fisher Scientific, Waltham, MA, USA) and manually scored using an Olympus IX 70 inverted fluorescence microscope with a CCD camera (Olympus, JPN) and CometScore 2.0 software (TriTek, Wilmington, DE, USA). Approximately 100 cells were randomly selected from each sample to assess DNA damage. The amount of DNA in the head (corresponding to low fragmentation) was evaluated.

### 4.7. Atomic Force Microscopy

HaCaT cells were seeded in Petri dishes (Willco, NLD) containing 2 mL of DMEM. The following day, the cells were exposed to UVA for 90 min and UVB for 20 min. Some cells were pre-incubated with 50 μM hyperoside for 4 h. Before irradiation, the growth medium was replaced with fresh DMEM. Immediately after irradiation, AFM measurements were performed using a Bioscope Catalyst atomic force microscope (Bruker, Billerica, MA, USA). The size of the cell scan was 100 × 100 μm and the scan rate was 0.2–0.3 Hz. For scanning, the silicon tip DNP-10-B on a nitride lever (spring constant of 0.12 N/m; resonant frequency of 23 kHz) was used.

### 4.8. Statistical Analysis

Data are presented as mean ± standard error for all measurements except for the Comet assay and atomic force microscopy, where the evaluation parameters correspond to the median and 25th and 75th percentiles. One-way analysis of variance (ANOVA) was used for comparisons between experimental groups. In the case of the Comet assay and atomic force microscopy, the Mann–Whitney U test using the software Statistica 13.4 software (TIBCO Software Inc., Palo Alto, CA, USA) was applied to evaluate differences in the DNA damage, cell diameter, and cell height. A *p* value of < 0.05 was considered statistically significant for all tests.

## 5. Conclusions

Our study focusing on the investigation of eight different UV-absorbing compounds on keratinocytes showed that hyperoside acts as an effective UV protective compound, especially against UVA radiation. This was also confirmed by an atomic force microscopy studying morphological changes in HaCaT cells or a study conducted on a 3D skin model. Common compounds used in sunscreens such as 2,4-dihydroxybenzophenone, 2-hydroxy-4-methoxybenzophenone, and 3-(4-methylbenzylidene)camphor turn out to be only physical UV filters, which may not have a direct and significant impact on cellular processes, especially at lower concentrations and short-term exposure to both the compounds themselves and UV radiation. According to the European Commission’s Scientific Committee on Consumer Safety (SCCS), 2-hydroxy-4-methoxybenzophenone (benzophenone-3 or oxybenzone) or 3-(4-methylbenzylidene)camphor can be used as a UV filter in sunscreen products at concentrations up to 6 or 4%, respectively [45,46]. UV filters penetrate the skin and enter the circulatory system. In the deeper skin layers, their concentrations reach approximately 2% of the applied doses after 6 h [47]. However, benzophenones as well as camphor derivatives are referred to as potential endocrine disruptors and therefore the FDA recommended that UV filters should be tested further for safety if their concentrations in plasma are greater than 0.5 ng/mL [48]. Conversely, pachypodol with a relatively large absorption in the UVA region appears to be not only toxic but also phototoxic. It is known for its antibacterial and antifungal effect. It also protects HepG2 cells from cell death caused by oxidative stress and attenuates ROS production by the activation of the Nrf2/ARE pathway [10]. However, in addition, it has been shown that it can inhibit the growth of the colon cancer CaCo-2 cell line [49]. Based on our results, we favor its cytotoxic potential rather than its cytoprotective role.

## Data Availability

The data that support the findings of this study are available from the corresponding author upon reasonable request.

## Supplementary Materials

The supporting information can be downloaded at: https://www.mdpi.com/article/10.3390/ijms24129910/s1.
