# Supplementary material for: Hyperoside as a UV Photoprotective or Photostimulating Compound—Evaluation of the Effect of UV Radiation with Selected UV-Absorbing Organic Compounds on Skin Cells
Source: Int J Mol Sci. 2023 Jun 8;24(12):9910. doi: 10.3390/ijms24129910 (PMC10298051; doi:10.3390/ijms24129910)
Supplement: Supplementary file 1 [file ijms-24-09910-s001.zip › ijms-2416531-supplementary.pdf]

Hyperoside as an UV Photoprotective or Photostimulating Compound—Evaluation of the Effect of UV Radiation with Selected UV-Absorbing Organic Compounds on Skin Cells

Anna Moukova [1], Lukas Malina [1,2], Hana Kolarova [1,2] and Robert Bajgar [1,2*]

[1] Department of Medical Biophysics, Faculty of Medicine and Dentistry, Palacky University in Olomouc, Hnevotinska 3, 775 15 Olomouc, Czech Republic; anna.moukova01@upol.cz (A.M.); lukas.malina@upol.cz (L.M.); hana.kolarova@upol.cz (H.K.)

[2] Institute of Molecular and Translational Medicine, Faculty of Medicine and Dentistry, Palacky University in Olomouc, Hnevotinska 3, 775 15 Olomouc, Czech Republic

* Correspondence: robert.bajgar@upol.cz; Tel.: +420-585-632106

Supplementary Materials:

Effect of UVA radiation on fluorescence of LysoTracker Blue DND-22

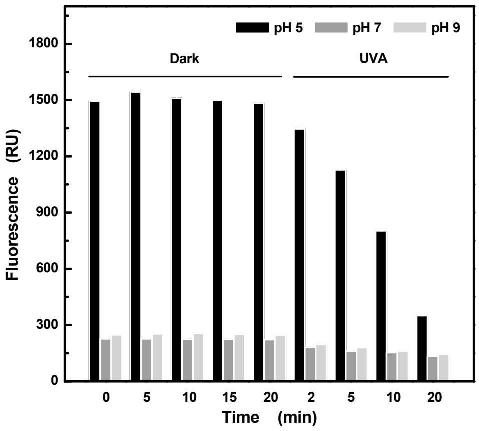

Figure S1. Dependence of LysoTracker Blue DND-22 fluorescence intensity on time of exposure to UVA radiation (at irradiance of 6.8 W/cm^2) and pH of PBS buffer

Spectral radiation distribution of the used UV sources

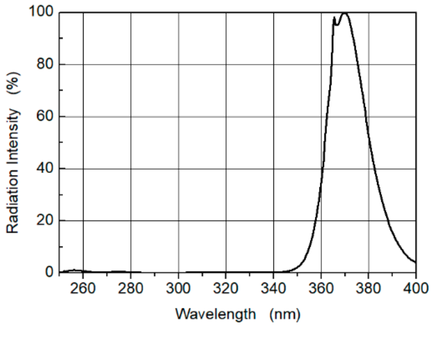

(a)

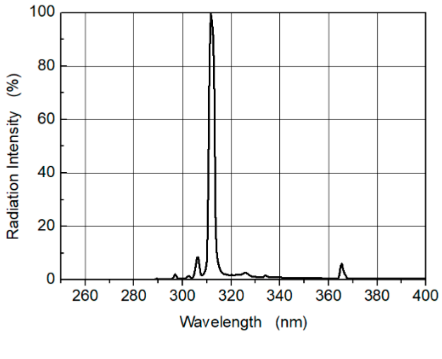

(b)

Figure S2. Spectral radiation intensity distribution of the used UV sources measured by the spectrofluorometer FLS980 (Edinburgh Instruments, UK): (**a**) broad-band UVA source; (**b**) narrow-band UVB source

Absorption spectra and chemical structures of UV-absorbing compounds

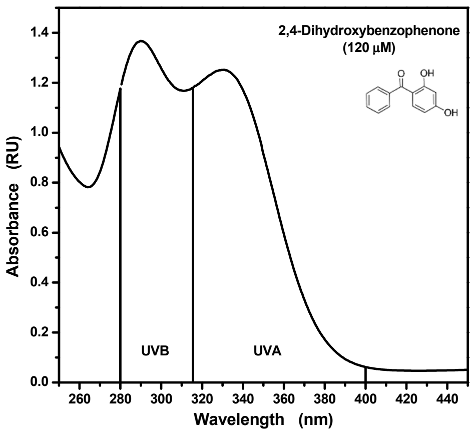

(a)

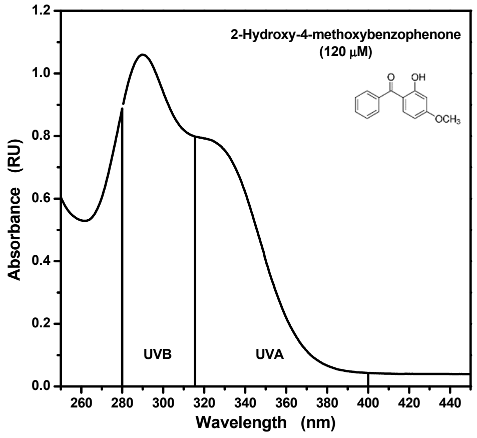

(b)

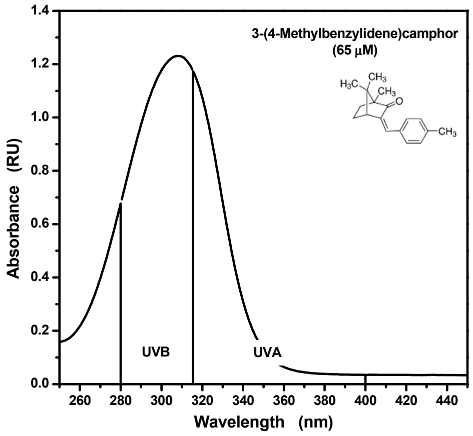

(c)

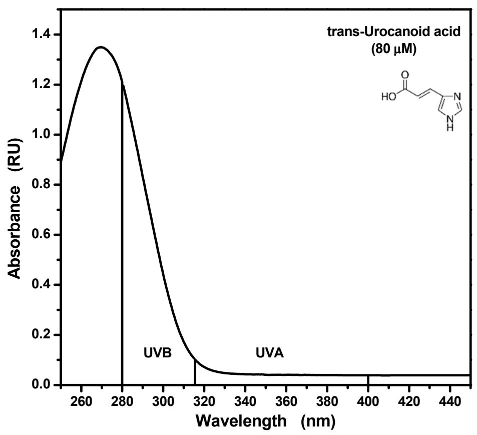

(d)

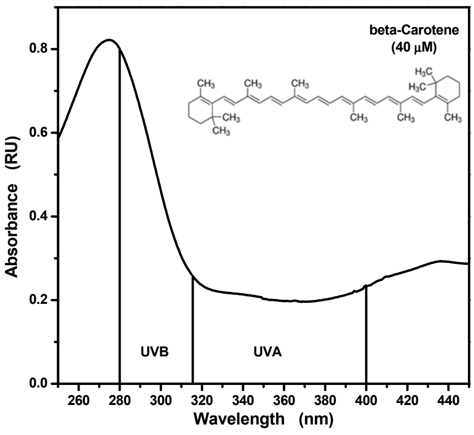

(e)

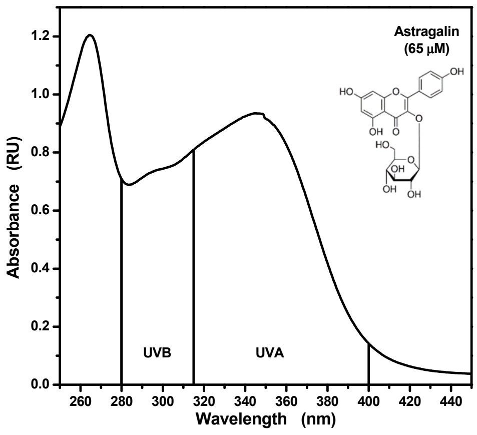

(f)

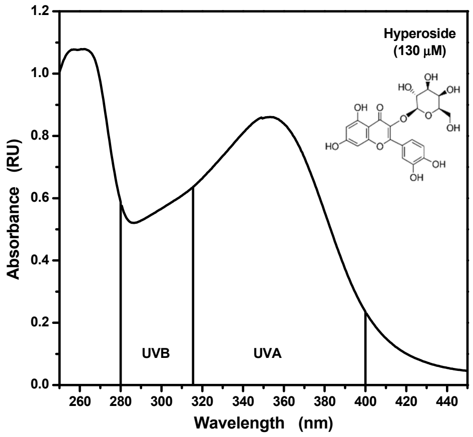

(g)

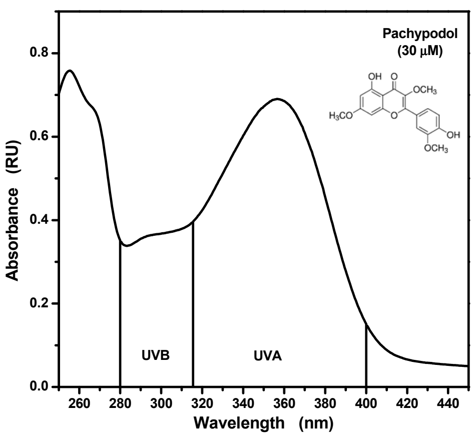

(h)

Figure S3. Absorption spectra of the analyzed UV-absorbing compounds: (**a**) 2,4-dihydroxybenzophenone; (**b**) 2-hydroxy-4-methoxybenzophenone; (**c**) 3-(4-methylbenzylidene)camphor; (**d**) trans-urocanic acid; (**e**) beta-carotene; (**f**) astragalin; (**g**) hyperoside; (**h**) pachypodol.
